# Pilomatrixoma of the Left Scapular Region in a Young Male: A Rare Case Report

**DOI:** 10.7759/cureus.15135

**Published:** 2021-05-20

**Authors:** Moeez Hassan, Nadia Altaf, Muhammad Waqas

**Affiliations:** 1 Pediatrics, Beaumont Hospital, Royal Oak, USA; 2 Pathology/Hematology, Khyber Medical University, Royal Oak, USA

**Keywords:** pilomatrixoma, benign neoplasm, skin, hair follicle, children

## Abstract

Pilomatrixoma is a benign and rare neoplasm derived from the cortex of the hair follicle. The head and neck region's involvement is relatively common and occurs in the upper extremity, trunk, and lower extremity in decreasing tendency. It is usually encountered in younger age groups, usually in children and adolescents. Management of the pilomatrixoma involves surgical excision of the mass. A 15-year-old male presented with a history of an isolated left scapular mass. The lesion had appeared two weeks before the presentation. His parents brought him to the emergency department due to continued bleeding from the mass. He denied any pain, fever, night sweats, weight loss, chills, appetite changes, and numbness to the area. Physical examination revealed a bleeding non-tender mass measuring about 2.5 x 3 cm, with the fluctuant inferior surface. Baseline investigations were normal. Cyst, abscess, and neoplasm were the initial differentials. Ultrasound findings were suggestive of neoplasm, and contrast-enhanced MRI was highly suspicious for progressive neoplasm. The surgical team was consulted, and surgical excision of the mass was carried out. Biopsy results confirmed pilomatrixoma. In conclusion, the pre-surgical diagnosis of pilomatrixoma is difficult. Hence, this case report's main objective is to raise awareness among clinicians about this rare diagnosis and its clinical features. Furthermore, our case highlights the need to consider pilomatrixoma in the differentials of the head, neck, and back masses in children and adolescents.

## Introduction

Pilomatrixoma is a benign, slow-growing rare tumor arising from the hair follicle matrix cells [1]. Mostly it is found on the head and neck region. Other sites include upper extremity, trunk, and lower extremity in decreasing order of tendency [2]. The tumor is mainly seen in the younger age groups, usually in children and adolescents. Surgical excision of the mass is the recommended treatment of choice [3].

Clinical suspicion must remain high, and physicians should use appropriate biopsy techniques to avoid misdiagnosis of pilomatrixoma [4]. Literature on pilomatrixoma is sparse. The objective of this case study is to raise awareness among clinicians about this rare disease and also to highlight its clinical features. Pilomatrixoma should always be considered in the differential diagnosis of a superficial mass in the children's head and neck region.

This case report was previously presented as a poster at APA Region 5 Conference-Pediatrics Post 2020-Looking Back & Looking Forward on March 5, 2021.

## Case presentation

**Figure 1:**
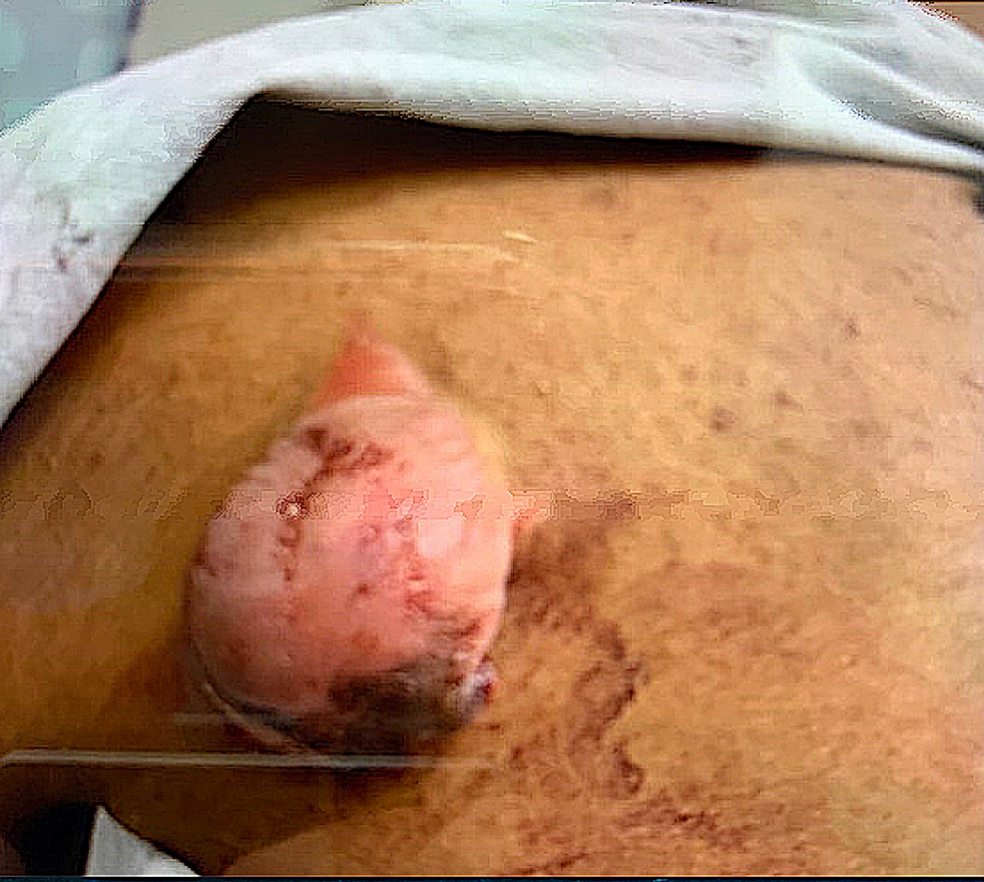
Left scapular mass Excoriations and scabs on the inferior surface of the mass.

**Figure 2:**
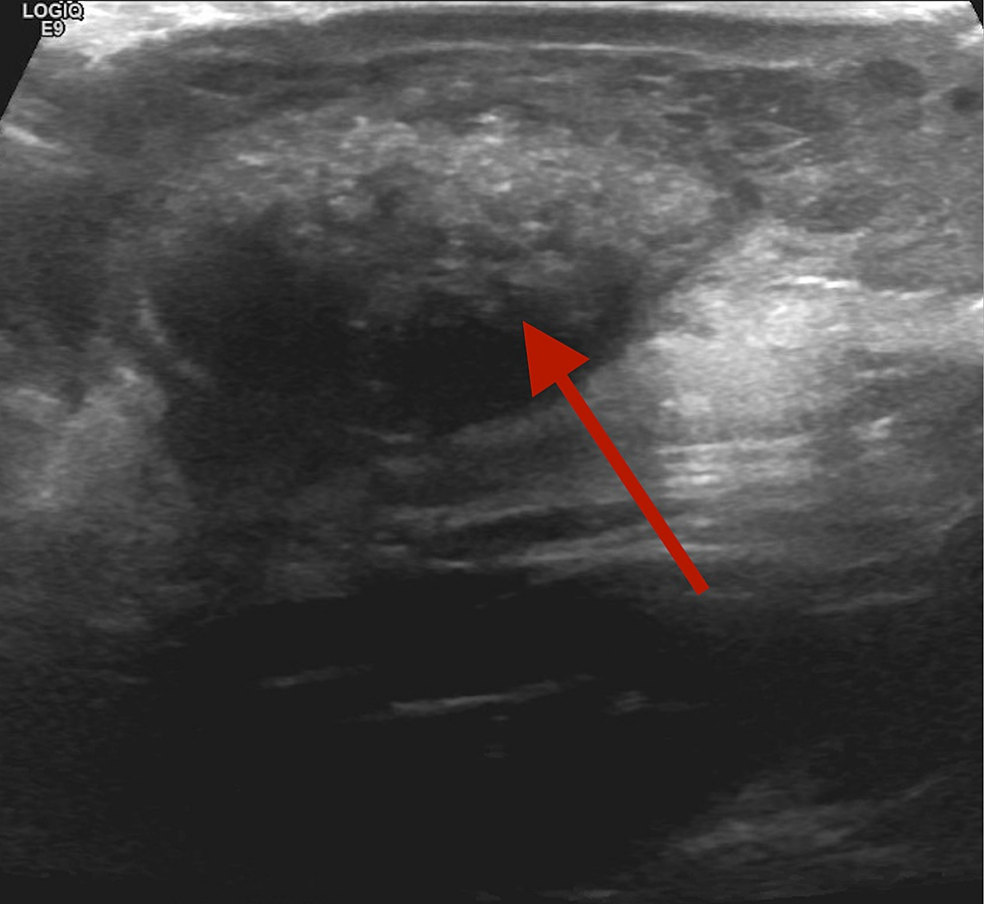
Ultrasound of the mass Ovoid heterogeneous mass involving the subcutaneous tissue of the posterior left shoulder with the associated crescentic hypoechoic area.

**Figure 3:**
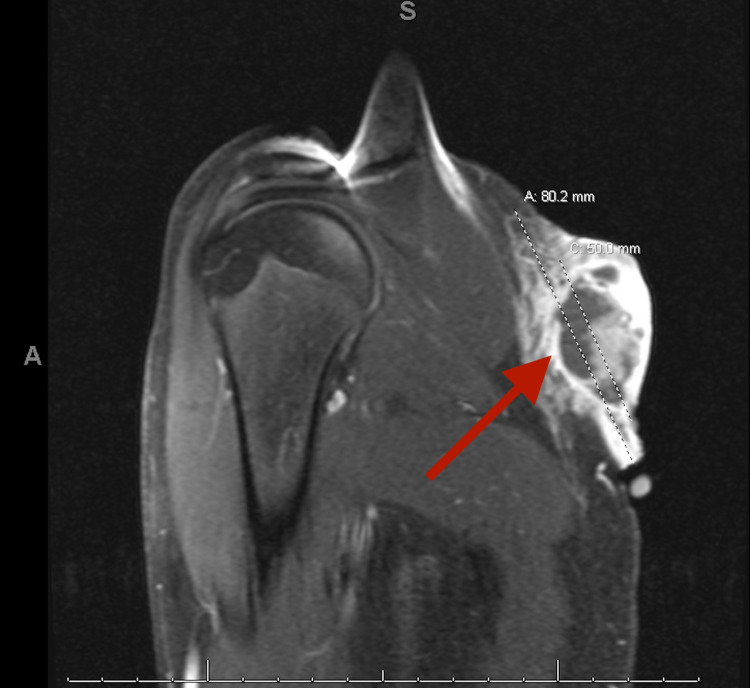
MRI of the mass Complex heterogeneously enhancing abnormality within the posterior upper left chest soft tissues with a multilobulated exophytic mass that protrudes posteriorly.

**Figure 4:**
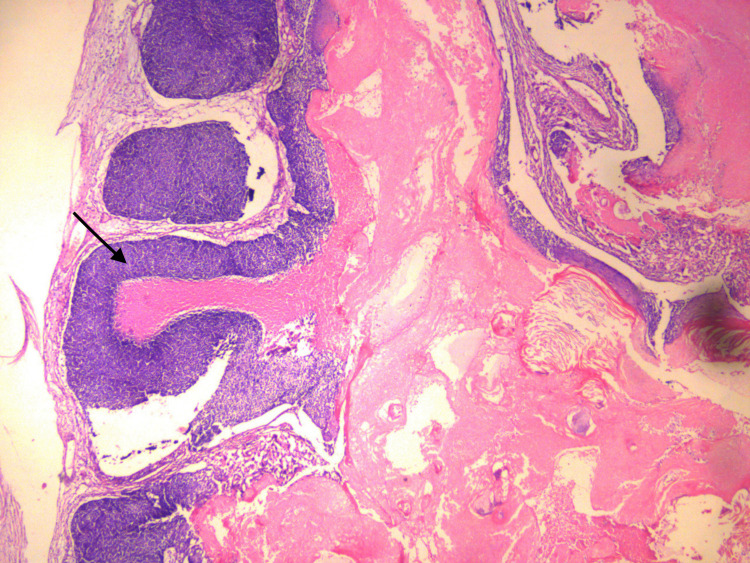
Section shows pilomatrixoma with both characteristic basaloid and eosinophilic cells (hematoxylin and eosin stain, x10) A solid mass of the basaloid component is seen in the early lesion. The well-defined demarcation seen between two characteristic components is due to the abrupt keratinization of the basaloid cells into eosinophilic (ghost) cells.

**Figure 5:**
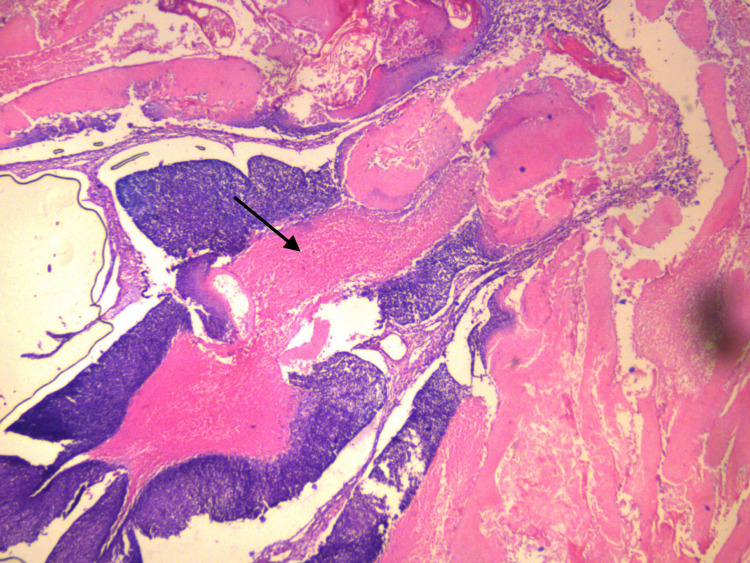
Section of pilomatrixoma showing both basaloid and ghost cells (hematoxylin and eosin stain, x10) The basaloid cells are replaced by structure less eosinophilic (ghost) cells with faint cellular outlines.

A 15-year-old previously healthy male with no significant past medical history presented to the emergency department for bleeding left scapular mass. He initially noticed the lesion two weeks before the presentation. The mass ruptured due to leaning against the wall. He was brought to the hospital by his parents due to continued bleeding. He denied any pain from the area except shortly after it ruptured. There was no history of fever, night sweats, weight loss, chills, appetite changes, and numbness to the site. Clinical examination revealed a non-purulent, solitary, and well-circumscribed mass located on the skin overlying the lateral side of the left scapula, measuring about 2.5 x 3 cm. On palpation, the mass was firm and non-tender with a fluctuant inferior surface. Excoriations and scabs were also noticed on the mass (Figure 1). The rest of the examination was unremarkable. Baseline investigations, including the complete blood count and basic metabolic panel, were normal. Ultrasound findings were suggestive of neoplasm, and contrast-enhanced MRI was recommended for further evaluation (Figure 2). Initial differentials included cyst versus abscess versus neoplasm. MRI findings were highly suspicious for progressive neoplasm (Figure 3). We consulted the surgical team, and the mass's surgical excision was carried out. Biopsy of the mass confirmed pilomatrixoma (Figures 4-5). On one year follow-up, no recurrence of the lesion was observed.

## Discussion

Pilomatrixoma is a slow-growing subcutaneous tumor arising from the cortex of the hair follicle [1]. Mostly, it is reported particularly on the head and neck region, and the lesion is solitary. Involvement of the upper and lower extremities is relatively rare. A review of 346 case series showed that 15.3% of pilomatrixoma was found in the upper extremities [5].

The lesion is usually encountered in younger age groups, usually in children and adolescents [3]. However, 40% of cases have been found in patients younger than 10 years of age, and 60% of cases occur before 20 years [6]. Surgical excision of the mass is the recommended treatment of choice because it does not regress spontaneously [7]. Recurrence after complete surgical excision is rare. Pilomatrixoma has a good prognosis. The malignant transformation in this lesion is rarely reported [8].

Pilomatrixoma is usually less than 3 cm in size. However, giant lesions of a size greater than 5 cm have been found. It is asymptomatic, firm, solitary, mobile, and well-circumscribed spherical to ovoid [9]. Pre-surgical diagnosis may be difficult in some cases. Pilomatrixoma should be considered in the differential diagnosis for head and neck masses in children. Dermoid cysts, sebaceous cysts, foreign body reaction, adenopathy, brachial cleft remnants, chondroma, preauricular sinuses, and giant cell tumors are the differential diagnoses of pilomatrixoma [10].

Pilomatrixoma is usually diagnosed on history and physical examination [11]. They are generally superficial, well-circumscribed, and small, due to which diagnostic imaging is not typically done. On clinical examination, the “tent sign” is pathognomonic for a pilomatrixoma. The lesion in this sign shows multiple facets and irregular angles on stretching the skin [12]. Incisional biopsy is the recommended diagnostic tool. Preoperative diagnosis can also be established on fine-needle aspiration cytology (FNAC), which usually shows an essential feature of pilomatrixoma such as basaloid cell, calcification, shadow (ghost) cell, and few nucleated squamous cells. Foci of calcification in pilomatrixoma are found on conventional radiography. Radiologic studies could be done to rule out malignancy and establish the spread of lesions [13].

## Conclusions

Pilomatrixoma is a rare subcutaneous neoplasm with a low recurrence rate, ranging from 0%-3%. The preoperative diagnosis of pilomatrixoma is difficult due to a lack of knowledge, and therefore, it is mostly misdiagnosed. The main objective of this case report is to raise awareness among clinicians about this diagnosis and highlight its clinical and radiological features. Pilomatrixoma is usually a benign tumor, but in sporadic cases, malignant transformation has been reported. The definitive treatment is the complete surgical excision of the mass. The clinicians may improve the presurgical diagnosis of pilomatrixoma by considering it in the differentials of the head, neck, and back masses in the pediatric population.
